# Prediction of the preoperative chemoradiotherapy response for rectal cancer by peripheral blood lymphocyte subsets

**DOI:** 10.1186/s12957-014-0418-0

**Published:** 2015-02-07

**Authors:** Noriko Tada, Kazushige Kawai, Nelson H Tsuno, Soichiro Ishihara, Hironori Yamaguchi, Eiji Sunami, Joji Kitayama, Koji Oba, Toshiaki Watanabe

**Affiliations:** Department of Surgical Oncology, Graduate School of Medicine, the University of Tokyo, 7-3-1 Hongo, Bunkyo-ku, Tokyo 113-0033 Japan; Department of Transfusion Medicine, Graduate School of Medicine, the University of Tokyo, 7-3-1 Hongo, Bunkyo-ku, Tokyo 113-0033 Japan; Department of Biostatistics, School of Public Health, Graduate School of Medicine, the University of Tokyo, 7-3-1 Hongo, Bunkyo-ku, Tokyo 113-0033 Japan

**Keywords:** Rectal cancer, Chemoradiotherapy, T lymphocyte, Peripheral blood

## Abstract

**Background:**

Although neoadjuvant chemoradiotherapy (CRT) has become a standard procedure to downstage locally advanced rectal cancer prior to surgery, markers to predict the response to CRT have not been fully identified. The aim of this study was to identify predictive factors of response to CRT, especially focusing on peripheral blood leukocyte subsets.

**Methods:**

A total of 45 consecutive patients diagnosed with primary rectal cancer were prospectively enrolled and received CRT followed by curative resection. The numbers of each lymphocyte subset in peripheral blood pre- and post-CRT were analyzed using flow cytometry. According to the pathological response to CRT, patients were classified into high (Hi-R) and low (Lo-R) response groups.

**Results:**

Hi-R cases had significantly higher numbers of pre-CRT lymphocytes (*p* = 0.018), T lymphocytes (*p* = 0.009) and helper T lymphocytes (Th lymphocytes, *p* = 0.015) compared to the Lo-R cases. With the receiver-operating characteristic curve for numbers of pre-CRT T lymphocytes, the area under the curve (AUC) was 0.733, and the optimal cutoff value was 1196/μl, with 76.5% sensitivity, 67.8% specificity, 59.1% positive and 82.6% negative predictive values. The numbers of pre-CRT Th lymphocytes and cytotoxic lymphocytes were both independent predictors of the high CRT response in the multivariate analysis.

**Conclusions:**

In addition to the direct cytotoxicity of CRT, recent studies have demonstrated the induction of an immunological host response, which also contributed to the tumor regression induced by CRT. Our result suggested the potential role of circulating T lymphocytes in predicting the response to CRT in colorectal cancer patients.

## Background

In the recent years, combined chemoradiotherapy (CRT) followed by total mesorectal excision has become the standard treatment for patients with locally advanced rectal cancer. Tumor downstaging with preoperative CRT increases the curative resection rate and lowers the local recurrence rate [1-3]. However, it is now evident that the preoperative CRT is not equally effective for all rectal cancer patients. Whereas some patients show an important regression with no detectable cancer cells in the primary tumor location or in lymph nodes in the surgical specimens, others have only a minimal response to CRT [4-6].

Although clinical factors such as gender, age or clinical TNM stage had little association with CRT response [7], many molecular predictors of tumor response to CRT have been determined, such as p53 [8], epithelial growth factor receptor (EGFR) [9,10], Ki-67 [11] and p21 [9]. We have also reported Ku70, Ku86, P16 and telomerase reverse transcriptase as promising biomarkers in predicting the radiosensitivity of rectal cancer [7,12,13]. Furthermore, we recently reported that the gene expression profiles of rectal cancer determined by DNA microarray analysis were correlated with the histological regression [14]. All these approaches are based on the idea that the chemoradiosensitivity of rectal cancer is closely associated with the direct cytotoxic effect of radiation by inducing DNA damage or apoptosis of cancer cells. However, growing evidence indicates the possibility that the tumor shrinkage of rectal cancer depends not only on the radiocytotoxity to cancer cells, but also on the tumor microenvironment and host immune response [15,16]. Recently we reported that both the density of tumor-infiltrating lymphocytes and those circulating in peripheral blood strongly correlate with the response rate of CRT in retrospective studies [17-19] and demonstrated the involvement of antitumor immunity in the cancer regression process induced by CRT.

In the present prospective study with new series of patients, we aimed to elucidate which subset of circulating lymphocytes most reflects the immune response evoked during CRT in patients with rectal cancer and analyze their predictive ability for the response to CRT.

## Methods

### Patients

A total of 45 consecutive patients diagnosed with primary rectal cancer of clinical T3-4 and M0 stage at the Department of Surgical Oncology, the University of Tokyo Hospital between January 2010 and June 2012 were prospectively enrolled in the study. The study protocol was approved by the local ethics committee (Research ethics committee, Graduate school of medicine and Faculty of medicine, The University of Tokyo), and written informed consent was obtained from all patients. All patients enrolled in the study received a total dose of 50.4 Gy of radiation and concomitant 5-FU based chemotherapy, and they underwent standardized curative resection, following an interval of 6–8 weeks after CRT. Barium enema (BE) was performed before and after CRT, and the longitudinal dimension of the rectal tumor was measured on BE images before (A) and after (B) CRT. The reduction rate was calculated as (A-B)/A, as described by Suzuki et al. [20,21].

### Pathological study

All of the resected specimens were examined pathologically, and the findings were recorded in accordance with the TMN classification. The histological regression of the primary rectal lesion in response to CRT was evaluated and classified as high or low, based on the amount of residual cancer according to the Japanese Classification of Colorectal Carcinoma, i.e., cases in which more than two-thirds of the cancer had degraded, necrotized or disappeared were classified as cases of high histological regression (Hi-R), and those with less than two-thirds reduction were classified as low histological regression (Lo-R).

### Blood sampling and analysis of leukocyte phenotypes

Peripheral venous blood samples were obtained before neoadjuvant CRT and 4–6 weeks after completion of CRT, prior to surgery. Samples were collected in ethylene diamine tetra-acetic acid collection tubes, and the blood cell counts in the samples were analyzed using an automated hematology analyzer (XE-5000, Sysmex, Japan). Lymphocyte subsets were also analyzed using flow cytometry, as described previously, with small modifications [22]. Briefly, whole blood was treated with FACS Lysing Solution (Becton Dickinson) to lyse red cells and fix leukocytes with 1% formaldehyde. Then, leukocytes were incubated with 10 μl of each antibody for 20 min at room temperature and analyzed in the flow cytometer. The region of lymphocytes was gated, and two-color flow-cytometric analysis for each cell phenotype on 10,000 events was performed on the FACSCalibur flow cytometer (Becton Dickinson) using the Multiset software package (Becton Dickinson), and the data were analyzed using the CellQuest software. A combination of isothiocyanate (FITC) and phycoerythrin (PE)-conjugated monoclonal antibody (Becton Dickinson, San Jose, CA, USA) was used to identify lymphocyte subsets, as follows: CD3(+)/CD19(−) for the T lymphocytes, CD3(−)/CD19(+) for the B lymphocytes, CD3(+)/CD4(+) for the helper T lymphocytes (Th lymphocytes), CD3(+)/CD8(+) for the cytotoxic T lymphocytes (Tc lymphocytes) and CD3(−)/CD56(+) for the natural killer cells (Table 1).

**Table 1:** **Definitions of lymphocyte subpopulations**

**Surface markers**	**Subpopulation**
CD3 (+)/CD19 (-)	T lymphocytes
CD3 (-)/CD19 (+)	B lymphocytes
CD3 (+)/CD4 (+)	Helper T lymphocytes (Th lymphocytes)
CD3 (+)/CD8 (+)	Cytotoxic T lymphocytes (Tc lymphocytes)
CD3 (-)/CD56 (+)	Natural killer cells

### Statistical analysis

The analysis of the associations between blood cell counts or numbers of lymphocyte subsets and clinicopathological variables was carried out using the Mann-Whitney *U* test. Association between the numbers of lymphocytes and tumor regression rate evaluated by barium enema was analyzed using the coefficient of correlation. Multivariate analysis with a logistic regression model was used in analyzing the independency of the predictors for CRT response. All analyses were performed with JMP 9 software, and *p*-values < 0.05 were considered to be statistically significant.

## Results

**Figure 1:**
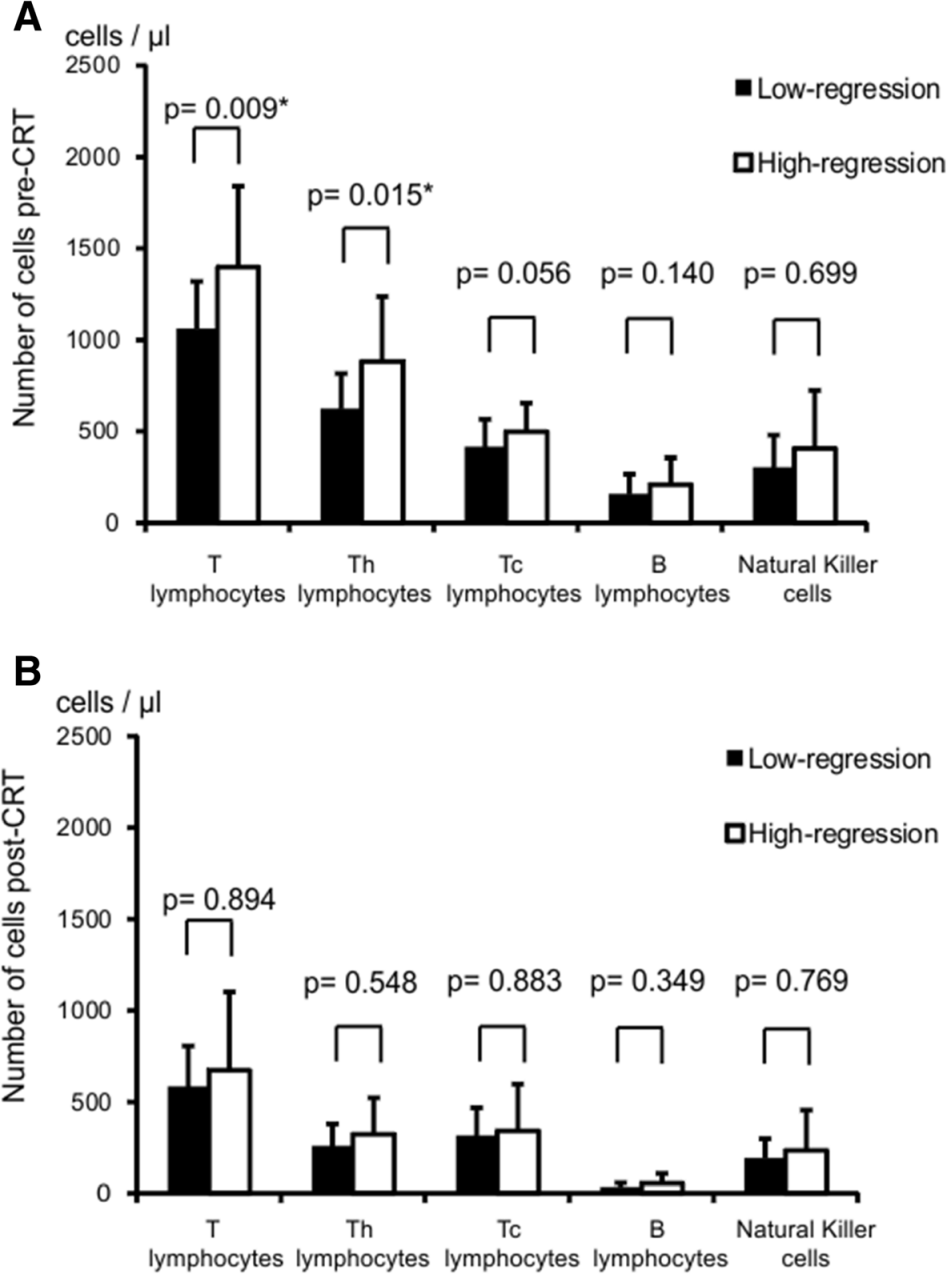
**The correlation between the pathological response and number of peripheral blood lymphocyte subsets before CRT.** *Filled bars* represent numbers of cells of pre-CRT **(A)** and post-CRT **(B)** in the Lo-R group and *blank bars* in the Hi-R group. Data are expressed as mean + SD of results. *Statistical significance.

**Figure 2:**
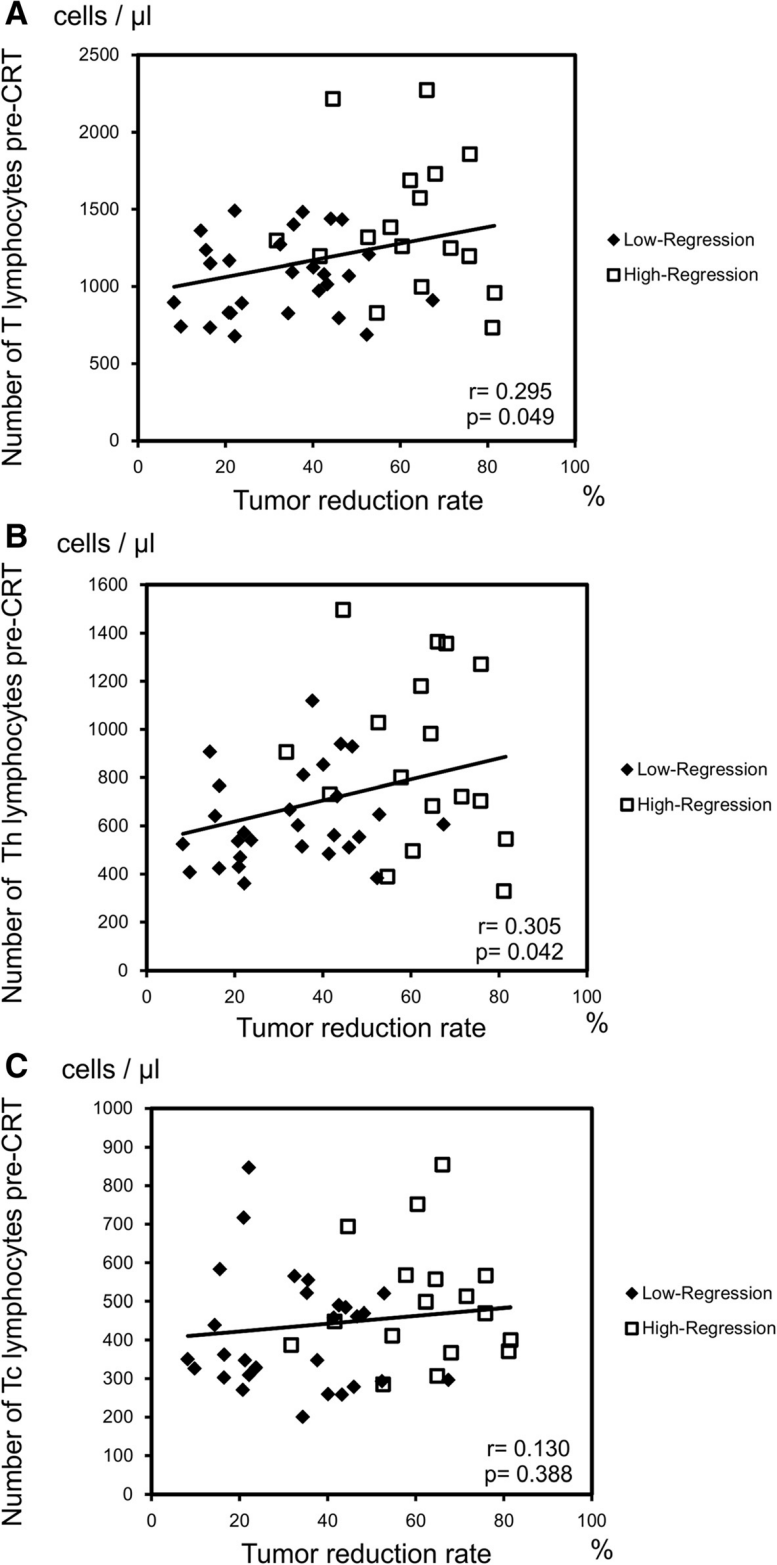
**The numbers of lymphocyte subsets before CRT and tumor reduction ratio in response to CRT.** The longitudinal length of the rectal tumor was measured by barium enema before and after CRT, and the tumor reduction ratio was calculated. Histological response grade was evaluated by the pathologists, according to the definitions in the Japanese Classification of Colorectal Carcinoma. The correlations between the numbers of T lymphocytes **(A)**, Th lymphocytes **(B)** and Tc lymphocytes **(C)** and tumor reduction rate are presented. Correlations of coefficient are presented as *r*.

**Figure 3:**
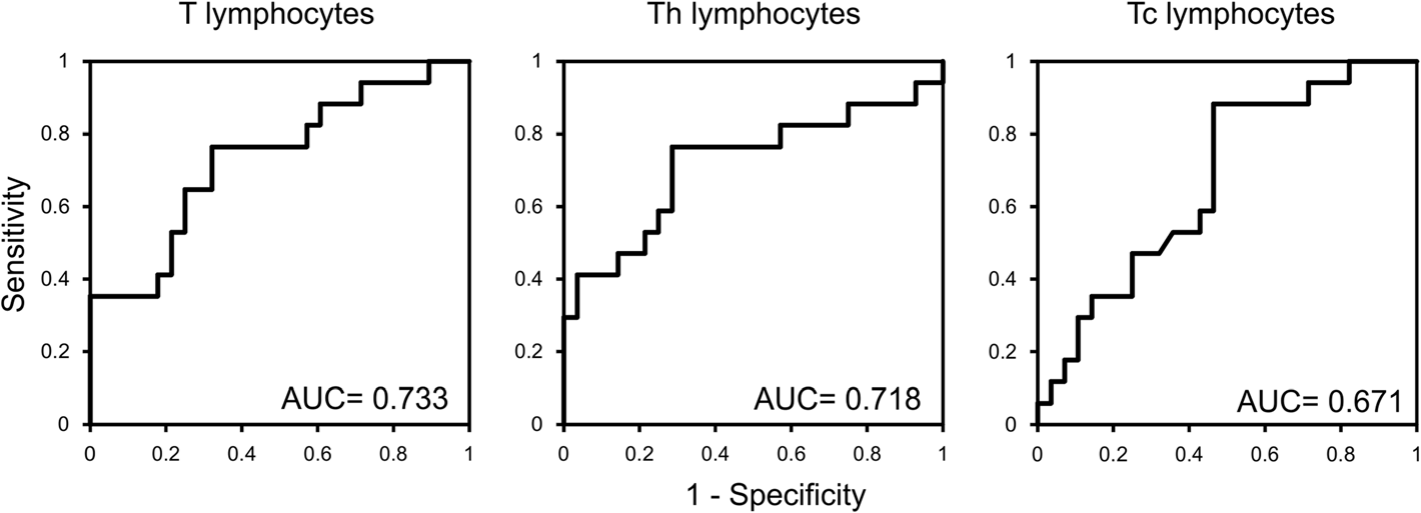
**The receiver-operating characteristic (ROC) curve for the numbers of pre-CRT T lymphocytes (A), Th lymphocytes (B) and Tc lymphocytes (C) in predicting the pathological high response group.** The *X-axis* represents “1–sensitivity” and the *Y-axis*, the sensitivity. The areas under the curve for each ROC curve are shown.

Patients’ characteristics are summarized in Table 2. The 45 patients were divided into two groups according to the pathological response, i.e., Lo-R (28 cases, 62.2%) and Hi-R (17 cases, 37.8%), and the 8 cases of pathological CR were included in the Hi-R group. The correlation between the pre- or post-CRT number of leukocyte subpopulations and the pathological response is presented in Table 3. Cases of Hi-R had significantly higher numbers of pre-CRT lymphocytes compared to the Lo-R, whereas in the analysis of post-CRT peripheral blood leukocytes, any subpopulation of leukocytes correlated with the pathological response. Therefore, we focused on the pre-CRT lymphocyte subsets and analyzed their correlation with the response to CRT. As shown in Figure 1A, the number of T lymphocytes and Th lymphocytes in Hi-R group was markedly higher than that in the Lo-R group (*p* = 0.009 and *p* = 0.015, respectively). Although without statistical significance (*p* = 0.056), the number of Tc lymphocytes also showed the same tendency. As expected from the results of the blood cell count, no difference between the Lo-R and Hi-R groups was observed for any lymphocyte subsets post-CRT (Figure 1B). Similar results were obtained in the analysis of the correlation between the number of lymphocytes and tumor reduction rate evaluated by BE (Figure 2). The pre-CRT total number of T lymphocytes (Figure 2A) and Th lymphocytes (Figure 2B) significantly correlated with the tumor reduction rate (*p* = 0.049 and *p* = 0.042, respectively), whereas the pre-CRT number of Tc lymphocytes (Figure 2C) and post-CRT lymphocyte subsets (data not shown) showed no correlation. The higher the number of pre-CRT lymphocytes, the higher the tumor regression achieved was. Figure 3 represents the receiver-operating characteristic (ROC) curve for numbers of pre-CRT T lymphocytes, Th and Tc lymphocytes in predicting the pathological response in the Hi-Response group. The area under the curve (AUC) was 0.733, 0.718 and 0.671, respectively. The optimal cutoff value of T lymphocytes was 1,196/μl, with 76.5% sensitivity, 67.8% specificity, 59.1% positive predictive value (PPV) and 82.6% negative predictive value (NPV). The cutoff value of Th lymphocytes was 683/μl, with 71.4% sensitivity, 76.5% specificity, 61.9% PPV and 83.3% NPV. The cutoff value of Tc lymphocytes was 367/μl, with 53.6% sensitivity, 88.2% specificity, 53.6% PPV and 88.2% NPV. Based on these cutoff values, each lymphocyte subset was divided into two groups, namely lower and higher, and multivariate analysis of each factor in predicting CRT response was performed. As shown in Table 4, none of the pre-CRT clinical factors showed a correlation with the CRT response, whereas high pre-CRT Th and Tc lymphocytes were independent predictive factors of a good response to therapy.

**Table 2:** **Patient characteristics (** ***n*** **= 45)**

**Characteristics**		**Number of patients (%)**
Gender	Male	29 (64.4)
	Female	16 (35.6)
Age (years)	Median	64
	Range	43 − 81
Ratio of tumor reduction (%)		43.7 ± 20.4*
Histological regression	Low regression (Lo-R)	28 (62.2)
	High regression (Hi-R)	17 (37.8)
Depth of tumor	pT0-pT2	23 (51.1)
	pT3-pT4	22 (48.9)
Lymph node metastasis	Absent	32 (71.1)
	Present	13 (28.9)
Lymphatic invasion	Absent	38 (84.4)
	Present	7 (15.6)
Venous invasion	Absent	19 (42.2)
	Present	26 (57.8)
Histological classification	Well-differentiated adenocarcinoma	21 (46.7)
	Moderately differentiated adenocarcinoma	15 (33.3)
	Mucinous carcinoma	1 (2.2)
	No cancer remaining	8 (17.8)

Values are number (percentage) unless otherwise noted.

*Data are expressed as mean ± SD.

**Table 3:** **The relations between the pathological response and subpopulations of leukocytes, pre- and post- CRT**

**Pre-CRT**				**Post-CRT**			
	**Lo-R**	**Hi-R**	***P*** **value**		**Lo-R**	**Hi-R**	***P*** **value**
White blood cells (cells/μl)	6,250 (2,800 − 9,400)	6,500 (3,700 − 9,600)	0.150	White blood cells (cells/μl)	4,700 (2,300 − 7,300)	4,300 (2,200 − 9,100)	0.598
Neutrophils (%)	65.9 (53.2 − 79)	60.2 (45.2 − 72.9)	0.106	Neutrophils (%)	71.1 (51 − 85.5)	70.1 (53 − 79.8)	0.543
Neutrophils (cells/μl)	4,211 (1,652 − 6,476)	4,250 (1,898 − 6,257)	0.743	Neutrophils (cells/μl)	3,334 (1,403 − 6,071)	2,994 (1,540– 6,379)	0.440
Lymphocytes (%)	25.9 (16.1 − 38.2)	31.5 (15.2 − 42.4)	0.261	Lymphocytes (%)	18.5 (5.5 − 33)	17 (10.9 − 36)	0.935
Lymphocytes (cells/μl)	1,510 (896 − 3,487)	1,998 (1,183 − 3,331)	0.018	Lymphocytes (cells/μl)	801 (391 − 1,351)	663 (386 − 2366)	0.743
Monocytes (%)	5.1 (3 − 10.7)	5.5 (3.8 − 9)	0.535	Monocytes (%)	6.9 (3 − 11)	7.1 (3.4 − 13)	0.752
Monocytes (cells/μl)	289 (168 − 696)	374 (185 − 801)	0.198	Monocytes (cells/μl)	278 (171 − 748)	309 (132 − 663)	0.717
Eosinophils (%)	1.5 (0 − 5.1)	3 (0.5 − 7.6)	0.040	Eosinophils (%)	2.0 (0 − 8.9)	2.9 (0.3 − 10)	0.079
Eosinophils (cells/μl)	86 (0 − 418)	177 (31 − 669)	0.010	Eosinophils (cells/μl)	84.2 (0 − 543)	112 (27.3 − 450)	0.147
Basophils (%)	0.2 (0 − 1.3)	0.4 (0 − 1.3)	0.277	Basophils (%)	0.3 (0 − 2)	0.2 (0 − 1)	0.307
Basophils (cells/μl)	16.5 (0 − 122)	23.2 (0 − 107)	0.167	Basophils (cells/μl)	12.9 (0− 94)	11.8 (0 − 61)	0.357

**Table 4:** **Multivariate analysis of clinical factors in predicting CRT response**

		**Univariate**	**Multivariate**		
		***P*** **value**	**Hazard ratio**	**95% CI**	***P*** **value**
Gender	Male vs. female	0.9758			
Age	≦65 vs. >65	0.6547			
Clinical T	T3 vs. T4	0.8847			
Clinical N	N0 vs. N+	0.6547			
T lymphocytes	≦1,196 vs. >1,196 (cells/μl)	0.0033	1.46	0.18 − 14.6	0.7261
Th lymphocytes	≦683 vs. >683 (cells/μl)	0.0015	10.56	1.5 − 99.6	0.0124
Tc lymphocytes	≦367 vs. >367 (cells/μl)	0.0032	11.38	1.5 − 146.4	0.0194

**Figure 4:**
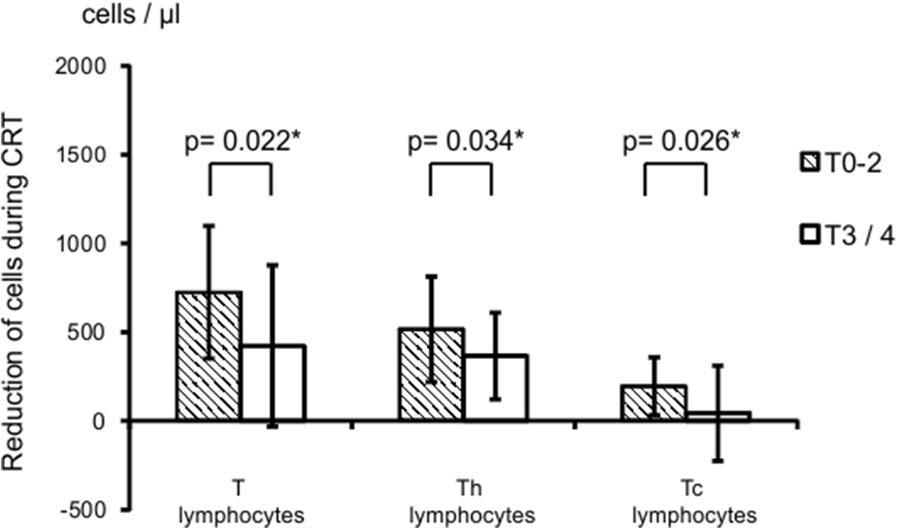
**The correlations between the reductions in numbers of lymphocyte subsets during CRT and pathological T factor of the resected specimens.** *Shaded bars* represent the reduction in numbers of each lymphocyte subset in the T0-2 cases and *blank bars* in the T3/4 cases. Data are expressed as mean ± SD of results. *Statistical significance.

We evaluated the association between lymphocyte subsets and the clinicopathological variables of the resected specimens. However, as presented in Table 5, both pre- and post-CRT lymphocyte subsets showed almost no correlation with the clinicopathological factors. Although the post-CRT number of B lymphocytes was higher in males than females (*p* = 0.026), and the number of natural killer cells in both pre- and post-CRT was higher in the cases with venous invasion (*p* = 0.024 and *p* = 0.018, respectively), these subsets account for only a small proportion of lymphocytes; thus, the clinical impact of these findings remains to be elucidated. The reductions in the number of T lymphocytes, Th and Tc lymphocytes during CRT are also shown in Figure 4. A significantly higher reduction in the number of T, Th and Tc lymphocytes during CRT was observed in the T0-2 than in the T3/4 group (*p* = 0.022, *p* = 0.034 and *p* = 0.026, respectively).

**Table 5:** **The correlations between the clinicopathological factors and numbers of lymphocyte subsets, pre- and post- CRT**

**Gender**	**Pre-CRT**			**Post-CRT**		
	**Male**	**Female**	***P*** **value**	**Male**	**Female**	***P*** **value**
T lymphocytes	1,196 (675 − 2,273)	1,046 (686 − 1,688)	0.325	608 (258 − 1,240)	455 (321 − 1,856)	0.228
Th lymphocytes	667 (329 − 1,489)	587 (383 − 1,180)	0.619	248 (89 − 696)	196 (118 − 692)	0.334
Tc lymphocytes	448 (258 − 855)	374 (200 − 847)	0.083	324 (119 − 671)	238 (137 − 1,144)	0.441
B lymphocytes	140 (31 − 675)	173 (30 − 409)	0.822	40 (1 − 185)	20 (9 − 73)	0.026
Natural killer cells	259 (56 − 966)	236 (135 − 1,020)	0.831	179 (15 − 811)	162 (35 − 497)	0.519
**Depth of invasion**	**Pre-CRT**			**Post-CRT**		
	**pT0-2**	**pT3/4**	***P*** **value**	**pT0-2**	**pT3/4**	***P*** **value**
T lymphocytes	1,196 (732 − 2,217)	1,157 (675 − 2,273)	0.489	482 (258 − 1,010)	694 (268 − 1,856)	0.136
Th lymphocytes	683 (329 − 1,496)	513 (361 − 1,364)	0.525	215 (89 − 620)	276 (99 − 696)	0.120
Tc lymphocytes	458 (200 − 847)	394 (270 − 855)	0.658	268 (141 − 495)	348 (119 − 1,144)	0.182
B lymphocytes	172 (73 − 675)	143 (30 − 459)	0.594	31 (1 − 140)	24 (9 − 185)	0.619
Natural killer cells	221 (135 − 966)	268 (56 − 1,020)	0.586	167 (28 − 335)	179 (15 − 811)	0.261
**Lymph node metastasis**	**Pre-CRT**			**Post-CRT**		
	**Absent**	**Present**	***P*** **value**	**Absent**	**Present**	***P*** **value**
T lymphocytes 1,135	(675 − 2,273) 1,233	(686 − 1,399)	0.507	494 (268 − 1,856)	628 (258 − 1,240)	0.332
Th lymphocytes	665 (329 − 1,496)	641 (383 − 907)	0.395	217 (99 − 696)	276 (89 − 564)	0.346
Tc lymphocytes	430 (258 − 855)	439 (200 − 752)	0.970	307 (119 − 1144)	316 (167 − 671)	0.324
B lymphocytes	178 (77 − 675)	122 (30 − 459)	0.089	26.5 (9 − 185)	30 (1 − 94)	0.769
Natural killer cells	260 (56 − 929)	232 (124 − 1,020)	0.590	173.5 (15 − 811)	174 (45 − 552)	0.837
**Lymphatic invasion**	**Pre-CRT**			**Post-CRT**		
	**Absent**	**Present**	***P*** **value**	**Absent**	**Present**	***P*** **value**
T lymphocytes	1,181 (732 − 2,273)	895 (675 − 2,217)	0.247	494 (258 − 1,856) 694	(366 − 1,240)	0.136
Th lymphocytes	675 (329 − 1,364)	602 (361 − 1,496)	0.491	210 (89 − 696)	276 (226 − 564)	0.100
Tc lymphocytes	453 (258 − 855)	350 (200 − 694)	0.434	268 (137 − 1,144)	348 (119 − 671)	0.206
B lymphocytes	167 (30 − 675)	122 (33 − 185)	0.103	27 (1 − 185)	30 (10 − 70)	0.931
Natural killer cells	232 (56 − 1,020)	495 (135 − 929)	0.199	167 (28 − 811)	201 (15 − 552)	0.283
**Venous invasion**	**Pre-CRT**			**Post-CRT**		
	**Absent**	**Present**	***P*** **value**	**Absent**	**Present**	***P*** **value**
T lymphocytes	1,122 (675 − 2,273)	1,181 (686 − 2,217)	0.774	481 (328 − 1,200)	619 (258 − 1,856)	0.227
Th lymphocytes	722 (329 − 1,364)	607 (383 − 1,496)	0.505	224 (146 − 696)	265 (89 − 692)	0.896
Tc lymphocytes	371 (200 − 855)	464 (258 − 847)	0.228	233 (119 − 495)	321 (137 − 1,144)	0.112
B lymphocytes	193 (73 − 409)	137 (30 − 675)	0.370	29 (10 − 185)	25 (1 − 121)	0.520
Natural killer cells	210 (117 − 498)	314 (56 − 1,020)	0.024	132 (15 − 811)	201 (80 − 552)	0.018

Data are expressed as median (range).

Pre-CRT: measured before chemoradiation therapy; post-CRT: measured after chemoradiation therapy.

## Discussion

In recent years, CRT has become one of the standard therapeutic modalities for the treatment of rectal cancer. Although CRT for rectal cancer has been shown to reduce postoperative local recurrence, it is now evident that preoperative CRT is not equally effective for all rectal cancer patients. If tumor response could be predicted before surgery, CRT-related toxicity and expense could be avoided for patients with resistant tumors, and/or more aggressive preoperative regimens could be considered for those patients. Therefore, a number of trials have been conducted to identify a useful marker for the prediction of the response to CRT [7-14]. Although most of the biomarkers identified so far are factors related to the radio-resistance of cancer cells themselves, in recent years, it has been elucidated that the immunological host response is also essential for tumor regression in CRT. Experimental T cell elimination remarkably reduced the therapeutic efficacy of radiation in a mouse model [23], and we have previously demonstrated that the densities of CD4(+) and CD8(+) tumor-infiltrating lymphocytes (TIL) were significantly associated with the histological grade after CRT, and the density of CD8(+) TIL was an independent prognostic factor for achieving CR after CRT [19]. Recently, we reported that a high pre-CRT lymphocyte count in the peripheral blood of rectal cancer patients was significantly associated with pathological response [18]. A similar result was also reported from Korea [24], but the subset of circulating lymphocytes mostly involved in the antitumor immunity evoked during CRT remains to be elucidated. Therefore, in this prospective study, we aimed to clarify whether circulating lymphocytes could reflect local immunological response and, if so, to identify the subset of lymphocytes mostly involved, which might be used as a potential predictor of the pathological CRT response.

Initially, we evaluated the association between peripheral blood leukocyte counts and the pathological response. Patients with high response showed a significantly higher pre-CRT number of circulating lymphocytes than those with low response, corroborating the results of previous reports [18,24]. Because the lymphocyte population assessed by the hematology analyzer contains several subsets, further analysis of lymphocyte subsets using flow cytometry was conducted. Pre-CRT circulating T lymphocytes and Th lymphocytes, but not B lymphocytes, showed a significant correlation with the pathological response and the tumor regression rate, and a high T lymphocyte or Th lymphocyte count correlated with a larger tumor reduction. Both T and B lymphocytes significantly decreased during CRT, and the post-CRT lymphocyte subsets did not differ between the high- and low-response groups. A number of precedent studies have documented the participation of T lymphocytes, but not B lymphocytes, in the radiation-induced antitumor immunity [16,25], and our present results were also concordant. The AUC of pre-CRT T lymphocytes in predicting the low-response group was as high as 0.733, and in multivariate analysis, the numbers of Th lymphocytes and Tc lymphocytes were independent predictors of response to CRT. Because the negative predictive values of each lymphocyte subset in predicting good response were higher than 80%, lymphocytes subsets should be a promising marker for the screening of CRT-resistant patients prior to CRT, thus avoiding unnecessary CRT. Some of the previously reported predictive factors of the CRT effect were based on the analysis of the surgically resected specimens; thus, their value as a predictive marker of CRT is low. For their analysis, biopsy specimens are necessary, which hold two problems: first, the findings obtained from post-CRT resected specimens may be affected by the CRT, not reflecting the primary nature of the tumor; second, the biopsy samples do not necessarily represent the whole cancer tissue because of the heterogeneity of cancer. In this respect, peripheral blood lymphocytes, which are easily obtained through the collection of a small amount of blood and represent the systemic immunologic condition, might have an advantage over these previously reported markers.

The correlation between lymphocytes in peripheral blood and tumor-infiltrating lymphocytes (TIL) has not been fully investigated. Milne et al. have reported that the lymphocyte count in peripheral blood did not correlate with CD8 (+) or CD20 (+) TIL in patients with ovarian cancer, and both lymphocyte count and TIL significantly correlated with prognosis, but the correlations were independent of each other [26]. Therefore, the role of circulating lymphocytes in antitumor immunity remains to be clarified; however, a number of reports have documented the association of a high peripheral lymphocyte count and favorable prognosis in patients with various types of malignancies including uterine, cervix, breast and nasopharyngeal carcinoma [27,28]. Our prospective study advanced these precedent findings in raising the following issues. First, peripheral lymphocytes seem to play a crucial role not only in treatment-naïve cancer tissue, but also in the CRT-mediated systemic anticancer immunological response. Second, among the various lymphocyte subsets, T lymphocytes, especially Th lymphocytes, seem to play a central role in the immunological response.

We also evaluated the association between the clinicopathological factors and the pre- and post-CRT peripheral lymphocyte subset counts. Contrary to the analysis of pathological regression, neither pre- nor post-CRT lymphocyte subsets showed a correlation with the clinicopathological factors. Interestingly, in cases with pathological T0-2, i.e., those with tumor downstaging, the higher reduction of T lymphocytes and Tc lymphocytes was induced by CRT. We previously reported that in vitro radiation-induced apoptosis of peripheral blood lymphocytes is correlated with histological regression of preoperative CRT [29], and the present finding of the correlation between the clinical reduction of lymphocytes induced by CRT and the downstaging of T factor strongly supports our previous finding.

Several problems, however, still remain to be addressed in our present study. Pathological regression strongly correlated with pre-CRT Th, but not Tc lymphocyte counts. In contrast, downstaging of the T factor correlated with post-CRT, but not with pre-CRT, Tc or Th lymphocyte counts. Cancer cells undergo apoptosis by the direct cytotoxic effect of radiation or chemotherapy, and consequently release cancer-specific antigens. These released antigens are phagocytized by antigen-presenting cells, such as macrophages or dendritic cells, resulting in induction of tumor-specific Th and Tc lymphocyte responses [30-32]. Furthermore, irradiated tissue releases various chemoattractant factors, such as CCL11 or CXCL16 [33,34], and promotes infiltration of lymphocytes into the cancer tissue. A more detailed role of peripheral Th and Tc lymphocytes in these processes should be clarified in further investigations. Another problem is that the observation period of this study was relatively short, and there were only a few patients with postoperative recurrence; thus, the effect of lymphocytes on prognosis is still unclear. Further investigation with a larger number of patients and longer follow-up period is desired.

## Conclusions

Both systemic and local immunity may play important roles in antitumor immunity, and from our present data, the analysis of the systemic one may efficiently predict the response to neoadjuvant CRT. The circulating T lymphocyte and Th lymphocyte counts would be promising markers for the prediction of response to CRT.

### Consent

Patient consent was obtained for the study presented in the manuscript.

## Abbreviations

CRT: Chemoradiotherapy
BE: Barium enema
Hi-R: High histological regression
Lo-R: Low histological regression
Th lymphocytes: Helper T lymphocytes
Tc lymphocytes: Cytotoxic T lymphocytes
